# Carbon nanotubes: From disordered ensembles to van der Waals crystals

**DOI:** 10.1016/j.xinn.2025.101017

**Published:** 2025-07-03

**Authors:** Yi Chen, Zhichun Zhang, Jiajun Chen, Peiyue Shen, Zhiwen Shi, Guangyu Zhang

**Affiliations:** 1 Tsung-Dao Lee Institute, Shanghai Jiao Tong University, Shanghai 201210, China; 2 Key Laboratory of Artificial Structures and Quantum Control (Ministry of Education), School of Physics and Astronomy, Shanghai Jiao Tong University, Shanghai 200240, China; 3 Beijing National Laboratory for Condensed Matter Physics, Key Laboratory for Nanoscale Physics and Devices, Institute of Physics, Chinese Academy of Sciences, Beijing 100190, China; 4 Songshan Lake Materials Laboratory, Dongguan 523808, China

## Abstract

Carbon nanotubes (CNTs) have ignited worldwide excitement for their extraordinary electrical and mechanical properties. Yet, despite decades of effort, realizing the “dream material” for carbon-based electronics—a wafer-scale, perfectly aligned, single-chirality, densely packed array of semiconducting CNTs—has remained elusive. Here, we provide a perspective on the recent breakthroughs that are bringing this vision closer to reality. We highlight advances in both chemical vapor deposition (CVD) and post-growth assembly, culminating in the emergence of van der Waals (vdW) crystals of single-chirality CNTs self-assembled on atomically flat hexagonal boron nitride (hBN) surfaces. We discuss the fundamental mechanisms, device implications, and rich opportunities for interdisciplinary exploration in quantum physics, materials science, and electronics.

## Main text

Carbon nanotubes (CNTs) are one-dimensional tubular structures that can be thought of as rolled-up graphene sheets. Since their serendipitous discovery in the early 1990s, CNTs have fascinated researchers across physics, chemistry, and engineering, holding the promise of a new technological revolution in nanoelectronics. Their unique one-dimensional nature gives rise to remarkable properties: ballistic transport, high current densities, and tunable electronic bands. These features make CNTs not only fascinating for fundamental study but also highly promising for next-generation electronics and optoelectronics. Each individual CNT can carry an electric current of approximately 10 μA, and given their extremely small diameter (about 1 nm), this translates to an astonishing current-carrying capacity per unit area. However, practical devices often require a much higher total current than a single nanotube can provide. As a result, recent research has increasingly focused on devices incorporating CNT films or CNT arrays within the channel to achieve greater overall current flow.

The last decade has seen remarkable milestones in the development and application of CNT-based electronics. In 2013, researchers achieved a groundbreaking feat by constructing the first simple computer with logic circuits entirely built from CNT thin-film transistors.^1^ Although this prototype operated with a basic architecture and at relatively slow speeds—far from the sophistication and performance of commercial silicon chips—it nonetheless marked a pivotal moment, demonstrating that carbon-based electronic systems could be realized in practice. In 2019, scientists developed a more complex CNT-based processor capable of running a 16-bit system, demonstrating substantial progress toward commercial chip manufacturing.^2^ By 2020, researchers successfully fabricated wafer-scale CNT transistors using commercial equipment originally designed for silicon semiconductor processing. This breakthrough confirmed the compatibility of carbon-based chip manufacturing with existing silicon-based production lines, significantly advancing the practical application of carbon-based chips.^3^ This breakthrough not only demonstrated the feasibility of integrating CNT-based device fabrication into the existing silicon-based semiconductor industry but also greatly advanced the prospects for the practical deployment of carbon-based chips in mainstream electronics.

However, obtaining high-quality and pure-semiconducting CNT array films is a challenge. Theoretically, CNTs can be constructed with infinitely many chiralities—each defined by how graphene is rolled. Crucially, only two-thirds are semiconducting, while one-third are metallic—a direct consequence of their chirality. This diversity is both a blessing and a curse: while it promises designer materials, it complicates the fabrication of high-performance electronic devices, which require purely semiconducting, uniformly aligned CNT arrays. The holy grail—articulated by Nobel laureate Richard E. Smalley in 2001—is a perfect single crystal composed of perfectly parallel, closely packed, single-chirality CNTs.^4^ Such arrays would unlock the full potential of CNTs in digital electronics, optoelectronics, and quantum science. Yet, aligning CNTs at high density, ensuring single chirality, and eliminating metallic tubes have proved formidable.

At present, there are two main approaches: chemical vapor deposition (CVD) and post-growth assembly techniques.

CVD growth typically produces randomly oriented CNTs, necessitating precise control over growth conditions to achieve directional alignment and produce parallel arrays. This alignment is often induced during growth through external forces, such as controlled gas flow, applied electric fields, or the introduction of atomic steps and lattice structures. In 2015, a team led by Jin Zhang^5^ developed a “Trojan catalyst” method to grow CNT arrays with a remarkable density of up to 130 CNTs per μm. Later, in 2017, they further advanced this approach by engineering catalyst shapes to control the diameter distribution of CNTs and selectively enhance the proportion of specific chiralities within the arrays.^6^ However, these methods still fall short of achieving the selective growth of entirely semiconducting CNTs, and the electrical performance of devices based on these arrays remains suboptimal. While metallic CNTs can be selectively removed through etching processes, this introduces defects and reduces overall nanotube density.

Post-growth assembly begins with dispersing CNTs in surfactant solutions, followed by purification and alignment on substrates via methods such as Langmuir-Blodgett and Langmuir-Schaefer techniques, dielectrophoresis, slow vacuum filtration, and self-assembly on functionalized surfaces. Notably, in 2020, the dimension-limited self-alignment (DLSA) technique^7^ enabled the deposition of arrays with a density of >100 tubes per μm and a semiconducting purity of 99.9999%. However, even with DLSA, perfect parallel alignment and close packing remain out of reach. Residual surfactants and tube entanglement further degrade device performance.

Both CVD and solution assembly face inherent trade-offs. CVD struggles to eliminate metallic tubes and achieve dense, uniform packing. Solution assembly inevitably introduces surfactant residues and tube entanglement; it cannot achieve perfect parallelism or atomic-scale order. Neither approach, even after two decades of global effort, has fulfilled Smalley’s dream of a van der Waals (vdW) crystal composed of single-chirality, densely packed, and perfectly aligned CNTs.

**Figure 1:**
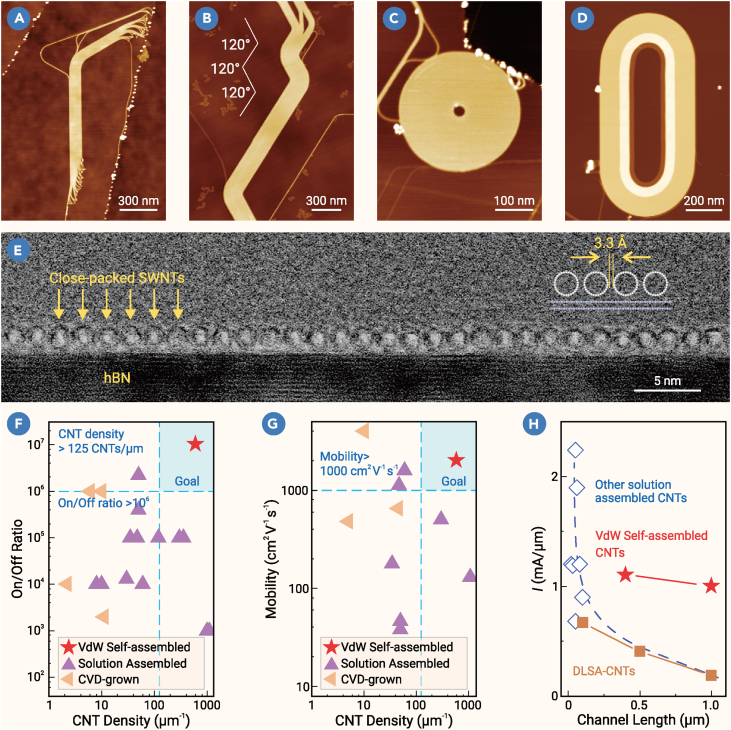
Morphology and device performance of carbon nanotube van der Waals Crystals (A–D) Atomic force microscopy (AFM) images of typical carbon nanotube (CNT) arrays. (E) Transmission electron microscopy (TEM) cross-sectional image of a typical CNT array on hBN substrate, where close-packed CNTs with uniform spacing can be clearly seen. The inter-tube distance of ∼3.3 Å is a result of inter-tube vdW interaction. (F–H) Benchmarking of our CNT array field-effect transistor (FET) results. Compared to the results found in existing reports, our homochiral CNT FETs (marked by red stars) exhibited superb on/off ratio, carrier mobility, and current-carrying capacity simultaneously. Adapted from Zhang et al.^8^ with permission.

A recent breakthrough surmounts these obstacles by harnessing atomically flat substrates—specifically, hexagonal boron nitride (hBN)—as platforms for CNT growth (Figure 1).^8^ The key insights are as follows: hBN offers atomic flatness, allowing the tubes to slide freely, while vdW interactions between CNTs facilitate their self-assembly during growth. CNTs nucleate and grow from catalysts. But as CNTs elongate, they glide across the hBN surface. When encountering hBN steps or other obstacles, the moving CNT bends and clings to the hBN step edges. Driven by growth forces and stabilized by vdW attractions with neighboring tubes, the CNT self-assembles into parallel, closely packed, single-chirality arrays—a true two-dimensional vdW crystal of CNTs.

Transmission electron microscopy (TEM) images reveal that the resulting films have a thickness of 1–2 nm (the diameter of a single CNT) (Figure 1E) and a tube density exceeding 600 tubes per μm—approaching the theoretical limit for a single-layer CNT film. Raman and Rayleigh spectroscopy confirm a single chirality across the array, while cross-sectional TEM images show perfect parallelism and tight packing via vdW forces. Crucially, theoretical analysis shows that this structure forms not by aligning many tubes but by one CNT folding and weaving upon itself multiple times—a process enabled by the superlubricity between CNTs and hBN. This explains the single-chirality nature and perfect alignment. This novel growth mechanism effectively explains the origin of single chirality in CNT arrays. Simultaneously, the vdW attractive interactions between the substrate and CNTs confine the sliding motion of CNTs to the planar direction, preventing inter-tube entanglement. It should be noted that the width of CNT arrays produced by this method is currently limited to several hundred nanometers, which restricts some applications. Nevertheless, this self-assembly mechanism provides new possibilities for creating novel vdW materials, and even at this scale, the CNT arrays exhibit impressive properties.

Benefiting from the unprecedented structural perfection, field-effect transistors (FETs) constructed from these arrays exhibit exceptional electrical performance: carrier mobility approaching 2,000 cm^2^V^−1^s^−1^, current-carrying capacity exceeding 6.5 mA per μm, and an on/off ratio as high as 10^7^. These device metrics not only surpass previously reported results for CNT-based systems but also exceed the projected benchmarks in silicon-based circuit roadmaps for future years (Figures 1F–1H). Notably, under identical channel lengths, the on-state current density of densely packed CNT array transistors significantly outperforms those fabricated by other methods. These outstanding characteristics highlight the immense potential of single-chirality, densely aligned CNT arrays for next-generation, high-performance carbon-based electronic chips.

Beyond electronics, the dense, defect-free arrays of CNTs provide a fertile platform for exploring novel physical phenomena. Theoretically, such arrays are expected to display pronounced anisotropy due to their structure. Moreover, strong electron correlations manifest as Luttinger liquid behavior, characterized by power-law transport and exotic collective phenomena. In dense, parallel arrays, inter-tube coupling introduces higher-dimensional correlated states, which are described by the sliding Luttinger liquid theory.^9^ Previous studies have hinted at the possibility of superconductivity in CNT bundles, where inter-tube coupling plays a crucial role.^9^ In perfectly ordered arrays, especially those with commensurate stacking between adjacent CNTs (as in arrays composed of armchair or zigzag CNTs), such coupling is enhanced, potentially giving rise to novel superconducting phases. For incommensurate stacking situations, manipulation of the electronic environment via electrostatic gating can tune the Fermi surface and induce momentum-matching conditions between adjacent CNTs, opening new pathways for realizing tunable quantum phase transitions and exploring rich inter-tube physics.

The quantum properties of electrons confined within CNTs are equally compelling. These include collective excitations and the phenomenon of spin-charge separation, hallmarks of one-dimensional quantum systems. In 2015, Zhiwen Shi, Feng Wang, and their colleagues conducted pioneering experiments on surface plasmon polaritons in single-walled CNTs.^10^ In CNT arrays, inter-tube coupling and dimensional effects lead to plasmonic resonances distinct from those observed in isolated tubes, thereby providing a new platform for the study and application of nano-plasmonics. The highly anisotropic optical properties of such arrays also enable the formation of hyperbolic plasmons analogous to those seen in hBN. Hyperbolic plasmons in CNT arrays are characterized by extreme spatial confinement, broad spectral tunability, and strong light-matter interactions, making them highly promising for advanced photonic and optoelectronic applications.

In summary, the emergence of perfect CNT vdW crystals represents a watershed moment, unlocking new research directions across multiple disciplines. In nanoelectronics, these materials provide an ideal channel for high-speed, low-power transistors, with enormous potential for integration and flexible electronics. In quantum materials, they serve as versatile platforms for probing correlated electron phenomena, topological phases, moiré superlattices, and artificial low-dimensional quantum matter. In photonics and plasmonics, arrays with controlled chirality and alignment offer tunable optical and plasmonic functionalities. For energy storage and sensing applications, the high surface area provides the potential for enhancing performance in batteries, supercapacitors, and ultrasensitive chemical and biological sensors. Looking ahead, critical challenges and opportunities remain: realizing wafer-scale production by extending vdW assembly methods, integrating *in situ* chirality selection with perfect alignment, harnessing and tuning collective quantum phenomena via inter-tube coupling, and ultimately fabricating large-scale, uniform, high-performance carbon-based circuits. The achievement of two-dimensional CNT vdW crystals not only fulfills a long-standing aspirational goal in materials science but also establishes a powerful new platform for fundamental discoveries and transformative technological innovations in carbon-based electronics and quantum materials.

## Funding and acknowledgments

This work was supported by the 10.13039/501100012166National Key R&D Program of China (no. 2021YFA1202900) and the 10.13039/501100001809National Natural Science Foundation of China (no. 12374292).

## Declaration of interests

The authors declare no competing interests.
